# Metabolic Reprogramming in the Tumor Microenvironment With Immunocytes and Immune Checkpoints

**DOI:** 10.3389/fonc.2021.759015

**Published:** 2021-11-11

**Authors:** Yaolin Xu, Lijie He, Qiang Fu, Junzhe Hu

**Affiliations:** ^1^ Department of Oncology, The People’s Hospital of China Medical University/The People’s Hospital of LiaoNing Province, Shenyang, China; ^2^ Department of Cardiology, The People’s Hospital of China Medical University/The People’s Hospital of LiaoNing Province, Shenyang, China; ^3^ The Second Clinic Medical College, China Medical University, Shenyang, China

**Keywords:** glycolysis, amino acid metabolism, lipid metabolism, nucleotide metabolism, mitochondrial biogenesis, the tumor microenvironment, immune checkpoints, PD-1

## Abstract

Immune checkpoint inhibitors (ICIs), Ipilimumab, Nivolumab, Pembrolizumab and Atezolizumab, have been applied in anti-tumor therapy and demonstrated exciting performance compared to conventional treatments. However, the unsatisfactory response rates, high recurrence and adaptive resistance limit their benefits. Metabolic reprogramming appears to be one of the crucial barriers to immunotherapy. The deprivation of required nutrients and altered metabolites not only promote tumor progression but also confer dysfunction on immune cells in the tumor microenvironment (TME). Glycolysis plays a central role in metabolic reprogramming and immunoregulation in the TME, and many therapies targeting glycolysis have been developed, and their combinations with ICIs are in preclinical and clinical trials. Additional attention has been paid to the role of amino acids, lipids, nucleotides and mitochondrial biogenesis in metabolic reprogramming and clinical anti-tumor therapy. This review attempts to describe reprogramming metabolisms within tumor cells and immune cells, from the aspects of glycolysis, amino acid metabolism, lipid metabolism, nucleotide metabolism and mitochondrial biogenesis and their impact on immunity in the TME, as well as the significance of targeting metabolism in anti-tumor therapy, especially in combination with ICIs. In particular, we highlight the expression mechanism of programmed cell death (ligand) 1 [PD-(L)1] in tumor cells and immune cells under reprogramming metabolism, and discuss in detail the potential of targeting key metabolic pathways to break resistance and improve the efficacy of ICIs based on results from current preclinical and clinical trials. Besides, we draw out biomarkers of potential predictive value in ICIs treatment from a metabolic perspective.

## Introduction

ICIs have shown impressive clinical anti-tumor performance in various cancers. Ipilimumab [commercialized anti-cytotoxic T-lymphocyte antigen-4 (anti-CTLA-4)] was the first approved ICI for treating patients with advanced melanoma (1, 2). In 2012, Topalian SL et al. reported a rather unexpected result, detailing that anti-PD-1 antibody produced objective responses in approximately one in four to one in five patients with non-small-cell lung cancer (NSCLC), melanoma, or renal cell cancer, with tolerable adverse events (AEs) (3). Subsequently, commercialized PD-1, Nivolumab and Pembrolizumab have been gradually applied in clinical anti-tumor treatment. In 2018, a study of IMpower133 demonstrated significantly longer progression-free survival (PFS) and overall survival (OS) in patients with small cell lung cancer (SCLC), which is traditionally considered a “recalcitrant cancer” (4), with atezolizumab (commercialized anti-PD-L1) being used based on traditional chemotherapy (etoposide combined with carboplatin) (5). Currently, ICIs alone or in combination with chemotherapy have been included in the NCCN guidelines for first-line, second-line and salvage therapy in numerous cancers, including recurrent or metastatic head and neck squamous carcinoma, advanced esophageal cancer, advanced non-epidermal growth factor receptor (*EGFR*)/anaplastic lymphoma kinase (*ALK*) mutated NSCLC, extensive-stage SCLC, triple-negative breast cancer (TNBC), advanced gastric cancer, advanced hepatocellular carcinoma (HCC), advanced colorectal cancer, advanced renal cancer, advanced urothelium carcinoma and postoperative or advanced unresectable or metastatic melanoma. However, the unsatisfactory response rates, high recurrence and adaptive resistance limit their benefits. Despite superiority to chemotherapy, a favorable response to Nivolumab in second-line therapy is still absent in approximately 80% of patients with non-squamous NSCLC (6). In TNBC patients, the response to PD-1 inhibitors is also relatively moderate (19%) (7). Consequently, many researchers have sought to investigate biomarkers with more predictive value, as well as additional therapy that could overcome the current therapeutic bottleneck of ICIs.

The metabolic reprogramming of cancer cells contribute to their transformation, tumorigenesis, tumor progression and immune escape under genetic or environmental orchestrating. The primary energy supply changes from oxidative phosphorylation (OXPHOS) to glycolysis, enriched amino acid metabolism, lipid metabolism and nucleotide metabolism, to accommodate the rapid growth demands of tumor cells during tumorigenesis and tumor progression. Deprivation of essential nutrients, altered metabolic processes and changed metabolites of cancer cells promote their transformation while influencing the recognition, activation, expansion and cytotoxic functions of tumor-associated immune cells to facilitate escape from immune surveillance. Moreover, metabolic reprogramming is also involved in the expression and downstream signaling pathways of immune checkpoint PD-(L)1. Compounds targeting metabolic reprogramming have been developed in anti-tumor therapy and exciting advancements have been reported in overcoming the limitations of immunotherapy. In this review, we attempt to describe the reprogramming metabolism of glucose, amino acid, lipid, nucleotide and mitochondrial biogenesis in tumor cells and immune cells and the roles they play in immune compromise, as well as the progress on targeting metabolism in combination with ICIs for anti-tumor therapy. In particular, we highlight the expression mechanism of immune checkpoint PD-(L)1 in tumor cells and immune cells under reprogramming metabolism, and discuss in detail the potential of targeting vital metabolic pathways to break resistance and improve the efficacy of ICIs based on results from current preclinical and clinical trials. We also draw out biomarkers of potential predictive value in ICIs treatment from a metabolic perspective.

## Immune Checkpoint With ICIs

Tumor cells, immune cells, metabolites, inflammatory factors, chemokines and other factors collectively construct the immunosuppressive TME. The immune checkpoint is one of the crucial factors participating in immune compromise, including co-inhibitory and co-stimulatory molecules. Tumor-associated or tumor-specific antigens are processed and expressed with major histocompatibility complex (MHC)-II by antigen-presenting cells (APCs). The processed antigens react with T cell receptor (TCR) expressed on T cells to provide the first stimulatory signal. There are also some ligands (such as CD28) expressed on T cells, reacting with their receptors, which act as co-stimulatory molecules to activate second stimulatory signals. After both signals are activated, T cells would proliferate, activate and serve cytotoxic functions. With a series of anti-tumor or anti-inflammatory cytokines [interferon-γ (IFN-γ), tumor necrosis factor-α (TNF-α), interleukin-1β (IL-1β)], or the inactivation of tumor suppressors (*PTEN* and *RB1)* (8, 9), the expression of co-inhibitory molecules up-regulates with the activation of toll like receptor (TLR)-/IFN-γ-mediated signaling pathways [nuclear factor κ-B (NF-κB), mitogen-activated protein kinase (MAPK), phosphatidylinositol 3 kinase (PI3K), mammalian target of rapamycin (mTOR) and janus kinase (JAK)] and downstream nuclear translocation of numerous transcription factors that transactivate expression of *CD274* gene (10–13), which alleviate the immune reaction and avoid further lethal effects, resulting in immune compromise in the TME. In addition to competing with co-stimulatory molecules to exert immunosuppression, co-inhibitory checkpoints also exert intrinsic immunosuppressive effects. When stimulated, PD-1 becomes phosphorylated at its immune receptor tyrosine-based inhibitory motif (ITIM) and immune receptor tyrosine-based switch motif (ITSM), which then bind the Src homology 2 (SH2) domains of SH2-containing phosphatase 2 (SHP2), initiating T cell inactivation (14). Protein phosphatase-2A (PP2A) mediates CTLA-4 suppression of T-cell activation by interacting with the cytoplasmic tail of CTLA-4 with the lysine-rich motif and the tyrosine residue (15). To resolve the immune compromise of immune checkpoints on immunocytes, compounds targeting inhibitory immune checkpoints are being studied and applied in clinical anti-tumor therapy.

Ipilimumab, an anti-CTLA-4 antibody, has demonstrated improved anti-tumor efficacy with enhanced immunity (1). Ipilimumab is the pioneer ICI applied in the clinical anti-tumor treatment and has acquired responses of approximately 20% (16). In the early stage of the activation of T cells, usually within 48-72h, CTLA-4 will be over-expressed. Co-inhibitory CTLA-4 competes with co-stimulatory CD28 to bind to CD80 or CD86 on the surface of APCs to alleviate the anti-tumor immunity of tumor infiltrated T lymphocytes (TILs), such as CD4+T, CD8+T cells and regulatory T cells (Tregs). Anti-CTLA-4 could partially relieve the immunosuppressive TME with enhanced anti-tumor immunity of T lymphocytes. Nevertheless, it is associated with a wide range of autoimmune disorders, including thyroiditis, diabetes mellitus, coeliac disease, myasthenia gravis, Addison disease, systemic lupus erythematosus, and rheumatoid arthritis when blockading CTLA-4 (17). These may be some of the reasons for the early and widespread immune-related side effects of Ipilimumab, along with improved anti-tumor immunity.

Similarly, PD-1 inhibits the proliferation and cytotoxicity of lymphocytes when ligated with its ligands PD-L1 (encoded by *CD274*) or PD-L2 (encoded by *PDCD1LG2*). Compared with Ipilimumab, Nivolumab (anti-PD-1 antibody) has a better prognosis with less frequent and more manageable immune-related AEs (18, 19). Anti-PD-1 and anti-CTLA-4 have distinct cellular mechanisms in checkpoint blockade. Anti-PD-1 predominantly induces the expansion of exhausted CD8+ TILs, while anti-CTLA-4 can induce the expansion of ICOS+CD4+ T helper 1 (TH1) effector lymphocytes in addition to exhausted CD8+ TILs (20).

In addition, other immune checkpoints such as T cell immunoreceptor with immunoglobulin and ITIM domains (TIGIT)/CD155 and T cell immunoglobulin mucin-3 (TIM-3)/Galectin-9 are coming to the forefront of researcher attention for their crucial roles in immune compromise. Co-inhibitory TIGIT and CD96 compete with co-stimulatory CD226 for their ligands CD155 and CD112 on dendritic cells (DCs) or tumor cells to suppress the anti-tumor immunity of TILs (21, 22). And the interaction between TIGIT and CD155 could transfer DCs into a tolerant state with increasing immunosuppressive cytokines (23). It has been reported that CD155 and TIM-3 are over-expressed on many tumor cells and negatively correlated with the survival time of most cancer patients (24–29).

Intriguingly, metabolic reprogramming is involved in inhibitory or stimulatory immune checkpoint downstream signaling pathways in immune cells to compromise their immune function. Lymphocyte activation initiates a program of cell growth, proliferation, and differentiation that increases metabolic demand for glucose uptake, glycolysis and glutaminolysis (30). Lacking large internal glycogen storage, with the help of cell surface receptors, resting lymphocytes are highly dependent on extracellular glucose import to meet increased metabolic needs. CD28 co-stimulation, acting through PI3K and Akt, is required for T cells to increase their glycolytic rate in response to activation. Nevertheless, co-inhibitory CTLA-4 inhibits Akt activation and blocks T cell activation to prevent the upregulation of glucose transport and glycolysis that results from CD28 costimulation (30). In addition to inhibiting aerobic glycolysis and glutaminolysis as caused by CTLA-4 ligation, PD-1/PD-L1 downward signaling increases fatty acid β-oxidation (FAO) in immune cells (31–33). The suppressed glycolysis impairs the differentiation of T cells to effectors, and enhanced FAO sustains the longevity of the exhausted T cells (33), which is likely to be regulated by the PD-1-leptin-signal transducer and activator of transcription 3 (STAT3) signaling pathway (34). Moreover, isotopomer analysis in primary human TILs further showed that PD-1 signaling resulted in increased reductive carboxylation of pyruvate, anaplerosis of the cycle at acetyl coenzyme A (acetyl-CoA) and succinyl-CoA, and block in *de novo* nucleoside phosphate synthesis accompanied by decreased rapamycin complex 1 (mTORC1) signaling (35). Furthermore, other costimulatory molecules (such as CD137) and cytokines are crucial regulators of metabolic reprogramming in TILs (36). As reported, the co-stimulatory receptor GITR supports CD8+ T cell proliferation and effector cytokine production by upregulating nutrient uptake, lipid storage, glycolysis and oxygen consumption rate (37).

Furthermore, glucose consumption by tumors metabolically restricts T cells, leading to their dampened mTOR activity, glycolytic capacity, and IFN-γ production. And checkpoint blockade antibodies against CTLA-4, PD-1 and PD-L1 could restore immune metabolic dysfunction of TILs with restored glucose, permitting CD8+ T cell glycolysis and IFN-γ production (38). Besides, Strauss L et al. demonstrated that PD-1 ablation assists emergency myelopoiesis and differentiation of effector myeloid cells to strengthen anti-tumor immunity by increasing intermediates of glycolysis, tricarboxylic acid cycle (TCA cycle), pentose phosphate pathway (PPP) and elevated cholesterol (39). Chang CH et al. demonstrated that blocking PD-L1 on tumors could dampen their glycolysis with decreasing glycolysis enzymes by inhibiting the Akt-mTOR pathway (38). Alternatively, by [18F]FDG-positron emission tomography (PET) and flow cytometry, Tomita M et al. demonstrated that anti-PD-1 treatment raises glucose metabolism in cancer cells by increasing glucose transporter 1 (GLUT1) and hexokinase-II (HK-II) expression, which may be implicated with inflammatory factors (such as TNF-α) secreted by active immune cells (40).

## Reprogrammed Metabolism of Glucose, Lipid, Amino Acid, Nucleotide and Mitochondrial Biogenesis in Cancer Cells With Immune Cells

With the orchestration of oncogenes and tumor suppressor genes, tumor cells not only re-edit their biological behaviors but also reprogram TME metabolism, which contributes to their invasion, migration, metastasis and even resistance to drugs (41, 42). Moreover, the reprogrammed metabolic processes of glucose, lipid, amino acid, nucleotide and mitochondrial biogenesis in tumor cells, and their altered metabolites, contribute to the evasion of immune surveillance and immune elimination (43–45). Metabolic competition in the TME is a driver of cancer progression (38) and immune compromise (46). The reprogrammed TME suppresses the immunity of anti-tumor TILs but supports the immunosuppressive TILs, thus sustaining tumor progression along with immune compromise. Besides, reprogramming metabolism in cancer cells and immune cells is one of the key obstacles in anti-tumor immunotherapy. There have been many attempts in targeting metabolic reprogramming to reinforce anti-tumor therapy, particularly in combination with ICIs.

## Hypoxia in the Immunosuppresive TME

Hypoxia, one of the crucial stressors in the TME, accelerates tumor cell progression and suppresses the immunity of TILs. The mismatch of rapid growth and proliferation of tumor cells with the lack of blood vessels contribute to hypoxia in the TME. Hypoxia-inducible factors (HIFs) play a vital role in this process. Under normoxic conditions, HIF-1α can be downregulated through ubiquitination and von Hippel-Lindau protein-mediated proteasomal degradation, while stabilization of HIF-1α and/or HIF-2α under hypoxic stress leads to transcriptional upregulation of many hypoxia-responsive genes associated with the metabolism and immunity of tumor cells and immune cells (47). Long-lasting HIF signaling could stimulate oncogene driven tumor progress, invasion, metastasis, angiogenesis, metabolic reprogramming, cancer stem cell maintenance and even constructing an immunosuppressive TME in various cancers (48, 49). Nevertheless, it was also reported that HIF-1α suppresses tumor cell proliferation in VHL-deficient renal cell carcinoma through the repressed aspartate-producing enzymes GOT1 and GOT2 and thus impaired oxidative and reductive aspartate biogenesis (50). HIFs promote lipid peroxidation and endoplasmic reticulum (ER) stress, recruit the immunosuppressive myeloid-derived suppressor cells (MDSCs) (51, 52), M2 macrophages (53) and Tregs (54) to construct an immunosuppressive TME. Besides, in hypoxic conditions, T cell expansion and cytotoxicity are suppressed due to impaired TCR (55). Moreover, HIFs also upregulate the expression of the inhibitory checkpoints [PD-L1 (56, 57), cluster of differentiation 47 (CD47) (58) and human leukocyte antigen G (HLA-G) (59)] to assist immune compromise. Oxygen is a crucial metabolite required for the TILs to differentiate appropriately upon PD-1 blockade (60). Hypoxia appears to be a significant metabolic barrier to immunotherapy, and remodeling the hypoxic TME may allow patients to switch from resistance to immunotherapy to gaining clinical benefit (61, 62). Accordingly, investigations of combining hypoxia-targeting drugs with ICIs have been studied. Metformin, a classic hypoglycemic drug for type II diabetes, can inhibit oxygen consumption by tumor cells and alleviate intratumoral hypoxia. Although metformin demonstrated modest anti-tumor effects, the combination of metformin with anti-PD-1 has been reported to significantly improve intratumoral T-cell function and tumor clearance in mouse models, and even in individual tumor patients (62, 63). Although metformin demonstrates immunosuppressive effects in models of graft-*versus*-host disease, lupus and graft rejection, it is preferentially taken up by tumor cells rather than T cells in the TME with the help of the organic cation transporter (OCT), which enhances T cell depredation on oxygen from cancer cells thereby boosting the immunity (62).

## Glucose Metabolism Reprogramming in Cancer Cells With Immune Cells and Their Effects on the Expression of Immune Checkpoints

Glucose metabolism is a significant supplier of energy and carbon sources in biological activities, including glycolysis (anaerobic oxidation), aerobic oxidation, PPP, glycogen metabolism, and gluconeogenesis. In the hypoxic TME, glycolysis is one of the critical metabolic reprogramming processes which could provide immediate and sufficient energy to satisfy the demand of tumor cells with rapid proliferation, adjusting to hypoxia environment and constructing an immunosuppressive TME. Tumor cell shifts metabolism to glycolysis, even in the presence of oxygen, which is called the Warburg effect (64). Hypoxia could activate multiple proglycolytic genes (*SLC2A1, LDHA* and *PDK1*) and glycolytic enzymes in favor of glucose uptake and glycolysis in both cancer cells and TILs, but depresses TCA cycle and aerobic oxidation (65–67). Lactate, the main metabolite of glycolysis involved in constructing an acidic TME, facilitates tumor progression and suppresses anti-tumor immunity through T cell suppression and PD-L1 upregulation (68).

**Figure 1:**
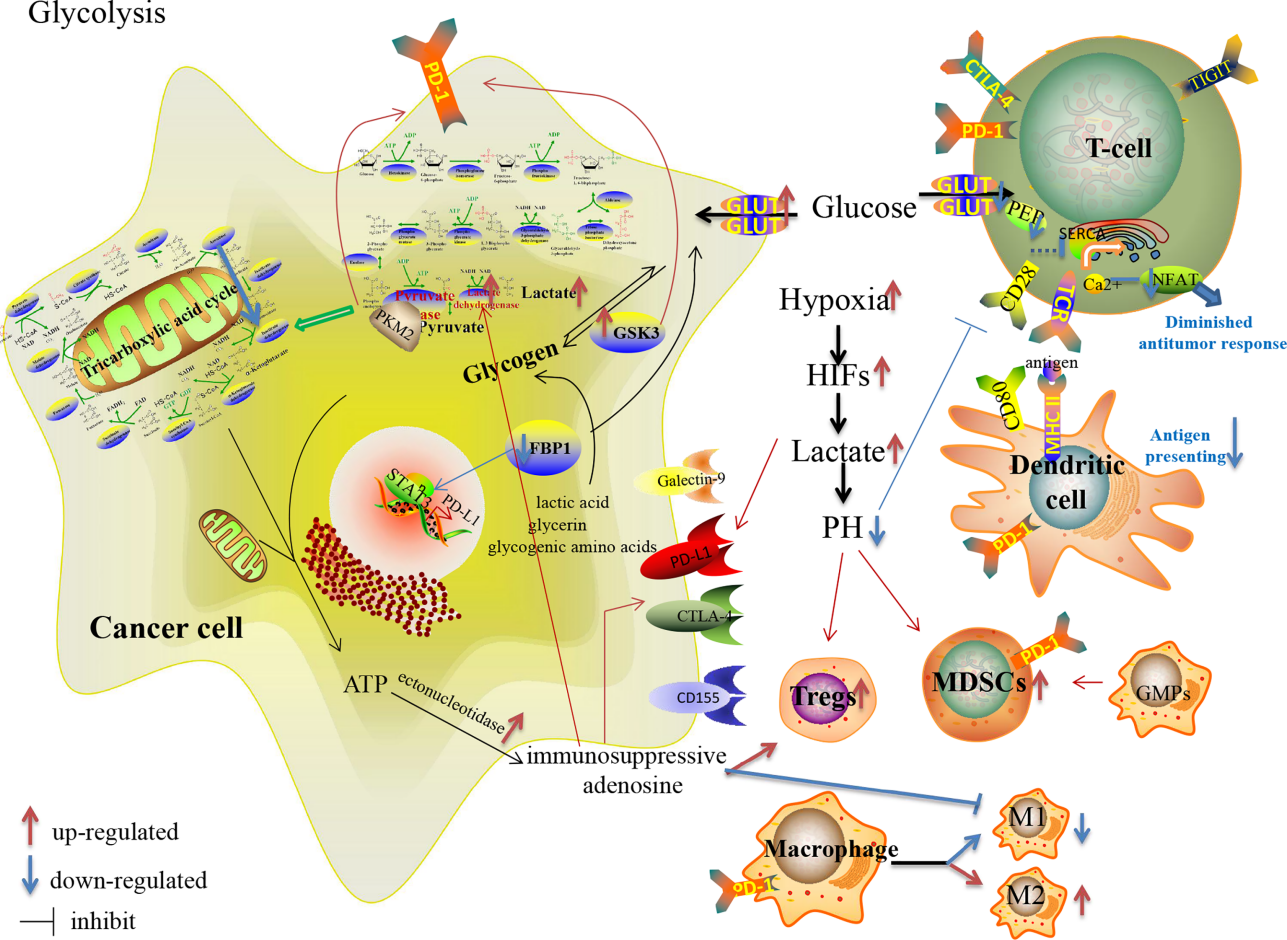
Glucose metabolism reprogramming in cancer cells and immune cells in the TME, and their effects on the expression of immune checkpoints.

The reprogrammed glycolysis in cancer cells and immune cells contributes to immune evasion. In the glucose-deficient TME, its key participants involved in anti-tumor immune regulation, CD4+TILs, CD8+TILs, MDSCs, M1/M2 TAMs, Tregs and other immune cells (**Table 1**), are dysfunctional due to metabolic reprogramming (69, 70) (**Figure 1**). Activated T cells engage aerobic glycolysis and anabolic metabolism for growth, proliferation and effector functions. High glucose and oxygen consumption by rapidly proliferating tumor cells inhibit TILs’ glycolytic activity by depriving them of nutrients, leading to dampened cytotoxic and effector function. Meanwhile, the decreased downstream metabolite phosphoenolpyruvate (PEP) is necessary for sustaining Ca^2^+-NFAT signaling in T cells, mediating diminished anti-tumor responses in glucose-poor TME (69). Besides, reprogrammed low pH in the TME leads to anti-tumor effectors anergy followed by apoptosis and engages with immunosuppressive MDSCs and Tregs (71). Moreover, pro-inflammatory M1 macrophages rely mainly on glycolysis and present two breaks on the TCA cycle that result in the accumulation of itaconate and succinate. Excess of succinate leads to HIF-1α stabilization that, in turn, activates the transcription of glycolytic genes, thus sustaining the glycolytic metabolism of M1 macrophages (72). Alternatively, the immunosuppressive M2 macrophages, which are more dependent on OXPHOS, may be dampened in the hypoxic TME.

**Table 1:** The metabolic reprogramming with different types of metabolism in immune cells in the TME.

Immune cells	Glucose metabolism	Amino acid metabolism	Lipid metabolism	Nucleotide metabolism
CD4+T cells CD8+T cells	Suppressed aerobic glycolysis in T cells leading to dampened cytotoxic and effector function.	Arginine and its metabolites are essential to TILs activation, and thus decreased arginine metabolism restricts innate and adaptive immunity.	CD8+ TILs uptake fatty acids mediated by increased CD36 participates in lipid peroxidation and ferroptosis, leading to impaired antitumor ability.	Required nutrient deficiency (one-carbon unit and aspartate) competing with cancer cells, impairs the immunity, such as activation of naive T cells and the expansion function of the effector T cell.
	Decreased PEP mediating diminished anti-tumor responses.	Tumor cells outcompete T cells for methionine, which disrupt the immunity.	Increased linoleic acid levels disrupt adaptive immunity specifically by depleting CD4+ T cells.	
	Reprogrammed low pH in the TME leads to antitumor effectors anergy followed by apoptosis.	With the upregulated IDO in tumor, both the depletion of tryptophan and the accumulation of kynurenine contribute to inhibiting effector T cell.	Enriched cholesterol accumulate in the cytoplasm with ER stress, inducing CD8+ T cell exhaustion with over-expression of inhibitory checkpoints.	
			Downregulated nSMase2 produces the decreased antitumor ceramide which impairs TH1 polarization and CD8+ TILs activation.	
Tregs	Reprogrammed low pH in the TME engages with Tregs.	Macrophages enhance the function of Tregs with secreted IL-23 in glutamine-addicted tumors.	SREBPs is upregulated in intratumoral Tregs, which are involved in enhanced expression of the PD-1 gene.	
		With the upregulated IDO in tumor, both the depletion of tryptophan and the accumulation of kynurenine contribute to stimulating Tregs.		
MDSCs	Reprogrammed low pH in the TME engages with MDSCs.	Both the depletion of tryptophan and the accumulation of kynurenine contribute to stimulating MDSCs.	Tumor cells derived GM-CSF induces FATP2 expression in MDSCs by STAT3 pathway activation, which confer the function of intratumoral PMN-MDSCs by the upregulation of arachidonic acid metabolism and the production of ROS.	
		MDSCs could block T cell activation by obstructing cystine and limiting the availability of cysteine in the TME.		
Macrophages	M2 macrophages more dependent on OXPHOS, may be dampened in the hypoxic TME.	αKG activates M2 macrophages by engaging FAO and epigenetic reprogramming of M2 genes.		
DCs		IDO over-expression in DCs suppress T cell-mediated immunity and activate Tregs with PD-1 expression.	Lipid accumulation restrain the tumor-associated antigen-presenting function of DCs with the lack of MHC and co-stimulatory molecules, so that they could not effectively stimulate T cells.	

Tregs, regulatory T cell; MDSCs, myeloid-derived suppressor cells; DCs, dendritic cells; PEP, phosphoenolpyruvate; TME, tumor microenviroment; OXPHOS, oxidative phosphorylation; TILs, tumor infiltrating lymphocytes; IDO, Indoleamine 2,3-dioxygenase; IL-23, interleukin-23; αKG, α-ketoglutarate; FAO, fatty acid oxidation; CTLA-4, cytotoxic T-lymphocyte antigen-4; ER, endoplasmic reticulum; TH1, T helper 1; SREBPs, sterol regulatory element-binding proteins; nSMase2, Neutral sphingomyelinase 2; PD-1, programmed cell death protein 1; GM-CSF, granulocyte macrophage-colony stimulating factor; FATP2, fatty acid transport protein 2; MHC, major histocompatibility complex; STAT3, signal transducer and activator of transcription 3; ROS, reactive oxygen species; IFN, interferon.

Moreover, many glucose-metabolizing enzymes involved in glycolysis, such as HK, 6-phosphofructokinase-1 (PFK-1), pyruvate kinase and lactate dehydrogenase (LDH), mediate the escape of tumor cells from immune surveillance. In addition to HIF-1α, the activities of these enzymes are regulated by the oncogenes *MYC* and *KRAS*, the tumor suppressor *TP53* and non-coding RNAs (73). The rate-limiting enzymes PFK-1, pyruvate kinase and HK play vital roles in glycolysis. Especially, pyruvate kinase isoform M2 (PKM2) is an essential participant in the processes of tumor progression and immune regulation, as well as glycolysis (74). Palsson-McDermott EM et al. reported that remodeled PKM2 up-regulated the expression of PD-L1 on tumor-associated macrophages (TAMs), DCs, T cells and tumor cells by regulating hypoxia response elements of Hif-1α target genes (75). Besides, in metabolism-reprogrammed TME, decreased expressions of GLUT and HK2 impair activated TILs, and these metabolic changes correlate with increased Tregs and expression of PD-L1 and Galectin-9 on cancer cells (76).

Increased expression of pH regulatory molecules, such as V-ATPase and carbonic anhydrase IX (CAIX)/CAXII, in the hypoxic TME, not only expel protons to sustain the intracellular homeostasis but also increase the immunosuppressive pressure mediated by the acidic metabolism (77). Neutralization of tumor acidity, such as bicarbonate, pH regulators and even RNAi nanoparticle, increases the infiltration with CD8+T and NK cells, decreases the number of immunosuppressive immune cells, and thus significantly inhibits the growth of tumors with potentiated anti-PD-1 therapy (78). Intratumoral acidosis and hypoxia may also dampen the bioactivity and distribution of ICI antibodies (79, 80). Zappasodi R et al. also suggested that the local glucose: lactate ratio may alter Tregs susceptibility to anti-CTLA-4, and decreasing tumor competition for glucose may facilitate the therapeutic activity of anti-CTLA-4 (81). Moreover, Shari Pilon-Thomas et al. reported that the combination of bicarbonate therapy with anti-CTLA-4 or anti-PD-1 improved anti-tumor responses in murine models (80). Besides, therapies targeting GLUTs (82) and LDH have been studied in anti-tumor therapy. Gong Y et al. reported that inhibition of LDH could enhance tumor response to anti-PD-1 immunotherapy in TNBC murine model (83).

In addition to glycolysis, glycogen metabolism and gluconeogenesis also reprogram the immunosuppressive TME. The process of glycogen synthesis is upregulated in many solid cancers, including renal, breast, bladder, uterine, ovarian, skin and brain cancers (84). Glycogen synthase kinase 3 (GSK3), encoded by *GSK3A* and *GSK3B*, is the crucial rate-limiting serine/threonine phosphatase in glycogen synthesis as well as PD-1 over-expression in cancer cells and CD8+T cells (85, 86). The downregulation or inhibition of GSK3 downregulates PD-1 expression in cancer (87). The use of GSK-3 inhibitors decreases PD-1 transcription by more than 80 percent, although the combination of anti-PD-1 with GSK-3 inhibitor could not further increase the killing of CD8+T cells (88). Gluconeogenesis converts non-glucose substances into glucose or glycogen. Fructose-1, 6-biphosphatase (FBP1), a negative regulator of glycolysis through inhibition of HIF-1α expression (89), is a critical enzyme in gluconeogenesis. It has been reported that FBP1 possesses a tumor suppressor function and it is often downregulated in many cancers, with the loss of FBP1 linked to tumor progression and poor prognosis in various cancer patients (89, 90). Bo Wang et al. further demonstrated that decreased FBP1 expression promotes tumor growth and resistance to ICIs in tumor-burdened mice with STAT3-PD-L1 over-expression, which provides a possible perspective for breaking therapeutic resistance to ICIs (91).

## Amino Acid Metabolism Reprogramming in Cancer Cells With Immune Cells and Their Effects on the Expression of Immune Checkpoints

**Figure 2:**
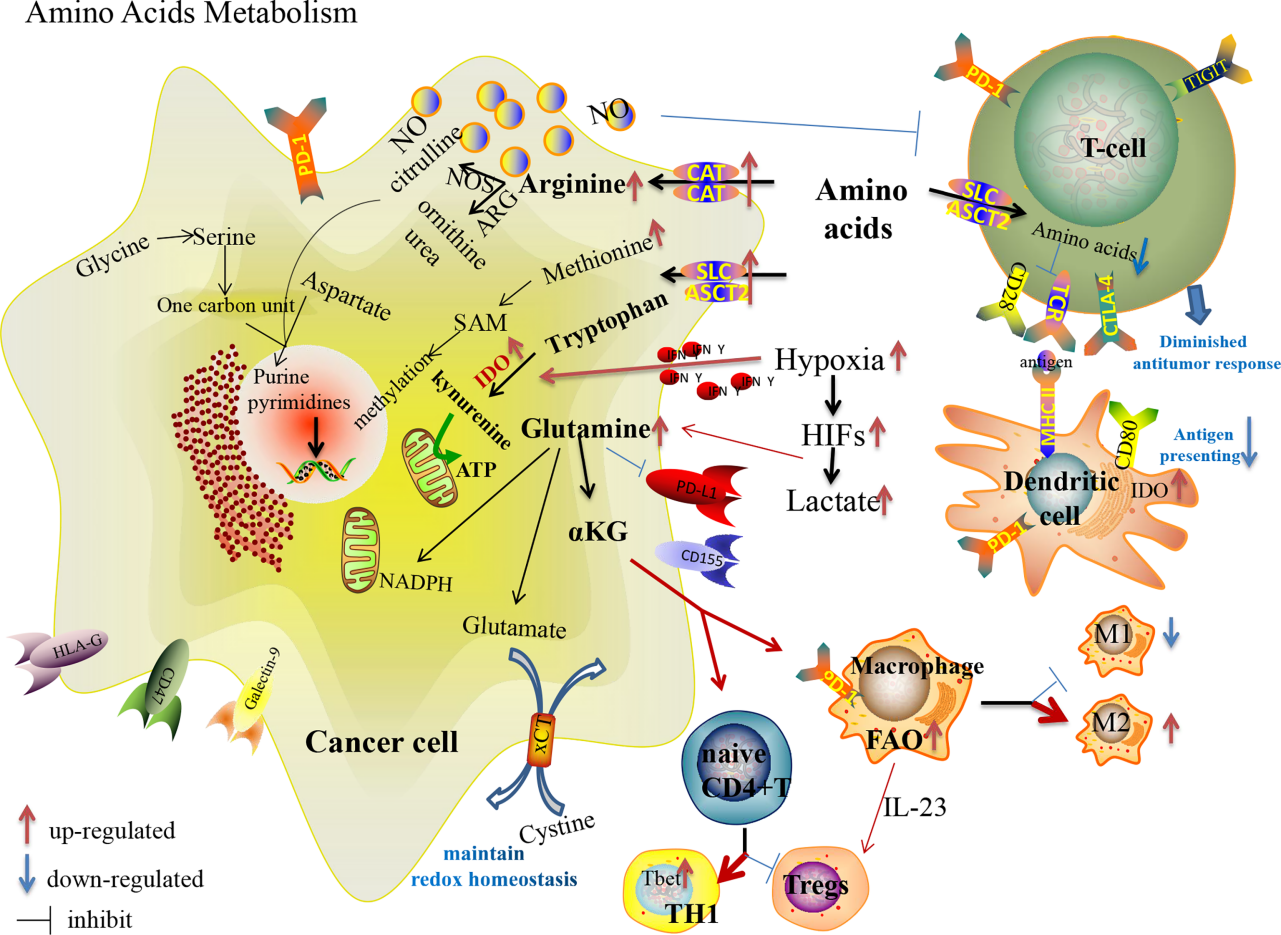
Amino acid metabolism reprogramming in cancer cells and immune cells in the TME, and their effects on the expression of immune checkpoints.

Apart from glycolysis and the Warburg effect, amino acids play critical roles in metabolic reprogramming to promote tumor progression in TME (**Figure 2**). Amino acids are avidly consumed to satisfy the demand of cancer cells proliferation with synthesizing proteins, nucleotides, hormones and neurotransmitters. Alternatively, the metabolism of amino acids is predominant in the activation and differentiation of T cells. When TCR signaling is activated, the expression of several amino acid transporters [solute carrier family (Slc7a5-Slc3a2), alanine, serine, and cysteine system amino acids transporter 2 (ASCT2)] are enhanced with T cell expansion and effector differentiation by activating mTOR (92). Nevertheless, the deprivation of essential nutrients from tumor cells dampens the function of immune cells (**Table 1**). Increasing attentions are focused on targeting reprogramming metabolism of amino acids, including glutamine, serine, glycine, arginine, tryptophan, methionine, cysteine and cystine, with increased clinical anti-tumor benefits.

Although glutamine is one of the non-essential amino acids (NEAAs), many tumor cells exhibit “glutamine dependence” in the TME with increased glutamine uptake and catabolism regulated with oncogene *KRAS* (93) and by lactate in a HIF-2α/c-Myc/ASCT2/glutaminase manner (94). Maintaining a high glutamine level provides enough carbon and nitrogen sources to support biosynthesis, bioenergetics and cellular homeostasis to drive tumor growth. Glutamine participates in macromolecular synthesis (amino acids, lipids, nucleotides, hexosamines, polyamines), signaling in cancer cells (95), vital support of the glutathione and nicotinamide adenine dinucleotide phosphate (NADPH) production, contributing to intracellular OXPHOS (96). Glutaminolysis supports proliferation and the integrity of the TCA cycle in tumor cells (97). Dysregulation of glutaminase and glutamine synthetase are vital events that allow anabolic adaptation of tumors (98). The increased glutamine metabolism in T cell activation regulates the skewing of CD4+ T cells toward more inflammatory subtypes, while the activation status of glutaminolysis during TCR-stimulation determines the activation of CD8+ T cells (99, 100). Consequently, the competition of cancer cells towards glutamine appears to suppress the immunity of TILs. Besides, macrophages enhance the function of Tregs with secreted IL-23 in glutamine-addicted tumors (101). As a crucial metabolite of glutaminolysis, accumulated α-ketoglutarate (αKG) has controversial effects on immunoregulation. On the one hand, αKG could activate immunosuppressive M2 macrophages by engaging FAO and epigenetic reprogramming of M2 genes (99); on the other hand, the activation of glutamine-deprived naive CD4+ T cells in the presence of αKG could induce the differentiation of TH1 cells instead of immunosuppressive Tregs with the increased expression of the gene encoding the TH1 cell-associated transcription factor Tbet and stimulation of mTORC1 signaling (102). In addition, glutamine metabolism is involved in the expression of PD-(L)1 in cancer cells and immune cells. Nabe S et al. reported that glutamine restriction enhanced anti-tumor cytotoxicity of CD8+T lymphocytes with decreased PD-1 expression and prevented CD8+T-cell exhaustion (103). Ma G et al. reported that the expression of PD-L1 in cancer cells upregulated during glutamine deprivation, and the upregulated PD-L1 restored to the normal level after glutamine recovery (104). Many compounds targeting glutamine metabolism and transition of glutaminase have been developed from preclinical to clinical trials (105), though there is a lack of impressive achievement, even when utilized in combination with ICIs.

Arginine metabolism is one of the important mechanisms responsible for tumor progression and immune suppression. Increased arginine metabolism can sustain the growth of many cancers while causing immunosuppression (106, 107). Arginine metabolism depends on the activity of the nitric oxide synthases (NOS) and arginases (ARGs) enzyme families. NOS oxidizes arginine into citrulline and NO, and ARGs hydrolyze arginine into ornithine and urea. After arginine enters the cancer cells through the membrane-bound transporters [cationic amino acid transporters (CAT1 and CAT2B)], it is metabolized by NOS to produce NO, which is associated with immune suppression, neovascularization and tumor progression (106). Arginine and its downstream metabolites (ornithine and citrulline) are essential to TILs activation, and thus deprivation of arginine from cancer cells restricts innate and adaptive immunity to further promote tumor survival and growth. Due to the lack of key argininosuccinate synthetase 1 (ASS1), some tumor cells cannot synthesize arginine by themselves. In this way, suppression of external arginine supply is regarded as a promising anti-tumor therapy. PEGylated arginine deiminase (ADI-PEG 20) has been applied to clinical anti-tumor therapy of HCC, melanoma and other ASS1-deficient cancers by depleting the external supply of arginine (108), which has also been shown to affect the hypoxia-induced processes by inhibiting HIF signal (109). However, one of the challenges of ADI-PEG 20 is drug resistance with re-expression of ASS1 or inhibitory immune checkpoints. Elena Brin et al. reported that the combination of ADI-PEG 20, anti-PD-1 and anti-PD-L1 results in a more powerful anti-tumor therapy in mouse models compared to monotherapy (110). A phase 1b clinical trial of ADI-PEG 20 combined with pembrolizumab demonstrated well-tolerated drug AEs and a promising disease control rate in patients with advanced solid cancers (111). Further clinical trials are needed in patients enriched for defined arginine auxotrophic cancers. Besides, another preclinical trial showed that the combination of protein arginine methyltransferase inhibitor with ICIs also enhanced the anti-tumor immunity with increased CD8+TILs (112). These researches provide potential strategies to overcome anti-PD-L1 resistance.

Similarly, tryptophan, one of the essential amino acids (EAAs), is also deprived in TME with rapid growth and progression of tumor cells. Indoleamine 2,3-dioxygenase (IDO), a vital enzyme involved in tryptophan catabolism with kynurenine production, is upregulated in many malignant tumors (113). The end-products of kynurenine are involved in the metabolic pathway of NAD+ and adenosine triphosphate (ATP) (114). Both the depletion of tryptophan and the accumulation of kynurenine contribute to the formation of an immunosuppressive TME by inhibiting effector T cell and NK cell functions, stimulating Tregs and MDSCs, and inducing polarization of macrophages to turn into a tolerogenic phenotype (46). Moreover, IDO-expressing APCs might induce systemic tolerance to tumor antigens (115). Interestingly, hypoxia initially leads to a lower IDO1/kynurenine expression in “tumor elimination stage” and increases IDO1 mRNA expression with activation of immune cells and secretion of cytokines (especially IFN-γ), thereby increasing IDO1/kynurenine expression to participate in “tumor escape stage” (116). Besides IFN signals, the ligation of CTLA-4 with CD80/CD86 was found to induce IDO expression in DCs, resulting in the suppression of T cell-mediated immunity (115). And IDO-expressing DCs could activate Tregs with PD-1 expression (117). Highly expressed IDO in immunocytes seems to be an obstacle in ICI anti-tumor therapy (118, 119). Rikke B. Holmgaard et al. found that the anti-tumor effect of anti-CTLA-4 or anti-PD-L1/anti-PD-1 was significantly increased in IDO-deficient melanoma-bearing mice (113), suggesting that the combination of IDO inhibitors with ICIs may enhance the anti-tumor ability in clinical therapy. Although the combination of IDO1 inhibitor (INCB024360) and pembrolizumab had encouraging results in phase I/II clinical trials of melanoma patients, it unfortunately failed to meet the endpoints in phase III trials (120). More clinical trials of this combination therapy for multiple malignancies are under investigation (121).

Sulfur-containing amino acids consist of methionine, cysteine and cystine, which can be converted into each other. Methionine, one of the EAAs, is the precursor of S-adenosyl methionine (SAM) which directly supplies the methyl in methylation. It is also involved in lipid metabolism by activating endogenous antioxidant enzymes such as methionine sulfoxide reductase A, and by glutathione biosynthesis to counteract oxidative stress. Cancer cells cannot proliferate when methionine is replaced with its metabolic precursor, homocysteine, while the proliferation of non-tumor cells is unaffected by these conditions, known as methionine dependence or the Hoffman effect (122). Growing evidences indicate that methionine restriction inhibits the growth of cancer cells and enhances the efficacy of chemotherapy and radiotherapy in preclinical and clinical trials (123). In addition to serving as a universal methyl donor, SAM has been reported to be essential for T cell activation and proliferation and has significant anti-tumor effects in numerous cancers, though there is insufficient SAM available in the TME (124). Recently, a preclinical trial on mouse model demonstrated that the combination of SAM and ICI can effectively block melanoma by alteration of key genes and pathways implicated in cancer and immune responses compared to monotherapy, providing the rationale for initiating clinical trials with SAM and ICI (124). By over-expressing the methionine transporter SLC43A2, tumor cells avidly consume methionine and outcompete T cells for methionine, which disrupts the immunity (125). Bian Y et al. demonstrated that the inhibition of SLC43A2 boosts checkpoint-induced tumor immunity with increased T cell immunity in tumor-bearing mice and patients with colon cancer (125). Cysteine is an EAA for T-cell activation due to the lack of cystathionase which converts methionine to cysteine. T cells also cannot import cystine and reduce it intracellularly to cysteine with a lack of an intact transporter (126). In this way, MDSCs could block T cell activation by obstructing cystine and limiting the availability of cysteine in the TME. Through the cystine/glutamate transporter cystine-glutamate exchange (xCT), the uptake of extracellular cystine is orchestrated in exchange for intracellular glutamate to maintain the redox homeostasis and promote tumor progression (127). The xCT inhibitors (such as sulfasalazine) exert anti-tumor effects through the production of reactive oxygen species (ROS), which induces cell death by disrupting redox homeostasis. Liu N et al. demonstrated the impact of inhibition of xCT on anti-PD-1/PD-L1 therapy in tumor-bearing mice. They showed that inhibition of xCT blunted the efficacy of PD-1/PD-L1 blockade through upregulating PD-L1 expression *via* the transcription factors IRF4/EGR1, and thus exosomes carrying large amounts of PD-L1 secreted from melanoma cells induced macrophage M2 polarization and eventually induced anti-PD-1/PD-L1 therapy resistance (127).

As one of the crucial NEAAs, serine is involved in the anabolism of multiple macromolecular substances by forming one-carbon unit, participating in purine and pyrimidine nucleotide synthesis, NADH/NADPH production and methylation pathways. Cancer cells obtain serine through four main pathways: Acquisition from the TME, degradation of cellular proteins, transamination of glycine, and *de novo* synthesis from glucose and glutamate. Enhanced serine uptake and synthesis in cancer cells can satisfy the demand for the rapid growth of tumor cells. Serine/glycine/one-carbon unit also affect the growth, proliferation and differentiation of immune cells (128, 129). As the main product of the one-carbon unit, NADPH is also involved in B cell proliferation (130).

## Lipid Metabolism Reprogramming in Cancer Cells With Immune Cells and Their Effects on the Expression of Immune Checkpoints

Lipids constitute the basic structure of membranes and function as signaling molecules and energy sources. Increased lipid uptake, storage and lipogenesis satisfy the demand of biological activities of rapidly proliferative cancer cells. Tumor cells could even synthesize new fatty acid by lipogenesis with activated fatty acids synthetase (FASN) which is partially controlled by mTORC2 (131), while normal cells can only uptake dietary fatty acid (132). Sterol regulatory element-binding proteins (SREBPs), a family of membrane-bound transcription factors in the ER, play a central role in regulating lipid metabolism and are linked to glucose metabolism by SREBPs cleavage-activating protein (133). Recent studies have shown that SREBPs are highly upregulated in various cancers and promote tumor growth (133).

**Figure 3:**
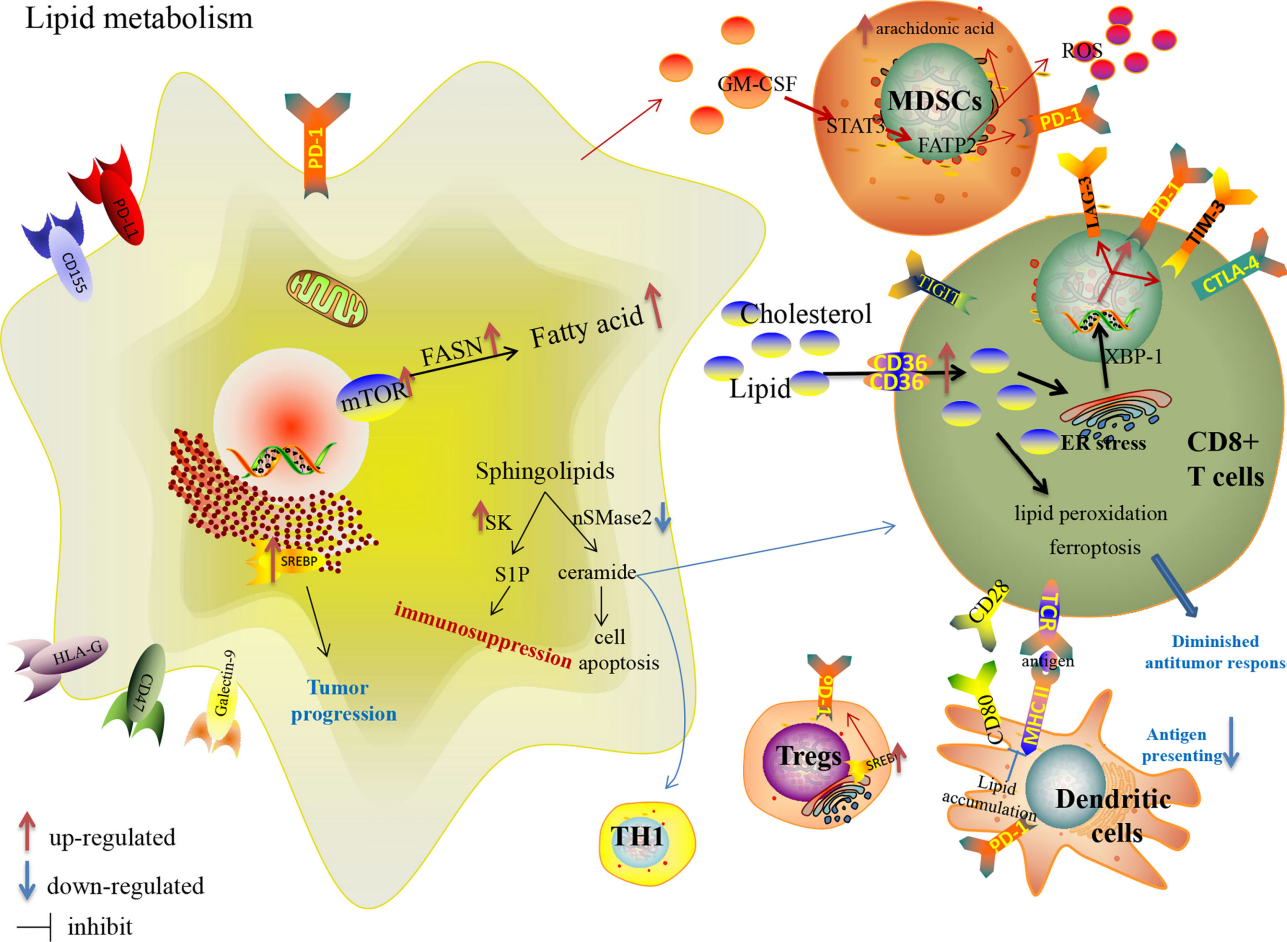
Lipid metabolism reprogramming in cancer cells and immune cells in the TME, and their effects on the expression of immune checkpoints.

Lipid metabolism in tumor-associated immune cells (**Table 1**) shapes an immunosuppressive TME favorable to tumor progression (**Figure 3**) (43, 134). A common metabolic alteration in the TME is lipid accumulation, a feature associated with immune dysfunction (135). Tumor cells derived granulocyte macrophage-colony stimulating factor (GM-CSF) induces fatty acid transport protein 2 (FATP2) expression in MDSCs by activation of STAT3 pathway, which confers the function of intratumoral PMN-MDSCs by the upregulation of arachidonic acid metabolism and the production of ROS (136). Adeshakin AO et al. reported that blockage of FATP2 expression in MDSCs by lipofermata decreased lipid accumulation, lower ROS, blocked immunosuppressive activity, down-regulated PD-L1 expression on CD8+ TILs, and even enhanced anti-PD-L1 anti-tumor efficacy in murine model (136). Fatty acid synthesis mediated by FASN contributes to the functional maturation of Tregs (137, 138). SREBPs are upregulated in intratumoral Tregs, which are involved in enhanced expression of the PD-1 gene (139). Inhibiting lipid synthesis and metabolic signaling dependent on SREBPs in Tregs unleash effective anti-tumor immune responses without autoimmune toxicity and even boost anti-PD-1 immunotherapy in murine model (139). Lipid accumulation restrain the tumor-associated antigen-presenting function of DCs with the lack of MHC and co-stimulatory molecules, so that they could not effectively stimulate T cells (43). Increased linoleic acid levels disrupt adaptive immunity specifically by depleting CD4+ T cells, which in turn promote carcinogenesis (140). CD8+ TILs uptake fatty acids mediated by increased CD36, which is induced by cholesterol in TME and participates in lipid peroxidation and ferroptosis, leading to decreased cytotoxic cytokine production and impaired anti-tumor ability in an oxidized lipid-CD36-p38 kinase manner (135, 141). Ma X et al. reported that blocking CD36 or inhibiting ferroptosis in CD8+ T cells effectively restored their anti-tumor activity and endowed greater anti-tumor efficacy in combination with anti-PD-1 antibodies in mice model (141). Rather than directly uptaking extracellular fatty acids in CD8+TILs, memory T cells rely on intrinsic mobilization fatty acids, engage FAO to a greater extent and support the metabolic programming necessary for development (142). Tissue-resident memory T cells (Trms), a subset of T-cells that produce higher amounts of cytokines than their circulating counterparts and provide enhanced local immunity, which is also associated with the success of ICIs therapy (143–146). Instead of utilizing glycolysis, Trms rely on FAO for cell survival, and deprivation of fatty acids results in Trms death (147). Targeting PD-L1 decreases fatty acid-binding protein (Fabp) 4 and Fabp5 expression in tumor cells, and the blockade of PD-L1 increases Fabp4/5 expression in Trms, promoting lipid uptake by Trm cells and resulting in better survival (147). However, a statistical study of large-scale datasets confirmed that tumors with activated lipid metabolism tend to have higher immune cells infiltration and better response to checkpoint immunotherapy (148). Harel M et al. also indicated that lipid metabolism is a regulatory mechanism that increases tumor immunogenicity by elevating antigen presentation and thus increasing sensitivity to T cell-mediated killing (149).

Cholesterol, a crucial component of membrane lipids, is required for TCR clustering and T-cell immunological synapse to participate in the antigen-presenting, activation and differentiation function of macrophages and DCs (150–152). It has been reported that as enriched cholesterol accumulates in the cytoplasm, it induces the over-expression of inhibitory checkpoints [PD-1, TIM-3, and lymphocyte activation gene 3 (LAG-3)] in an ER-stress-X-box binding protein 1 (XBP1)-dependent manner leading to the functional depletion of CD8+ T cells. Ma X et al. showed that decreased cholesterol or ER stress could enhance CD8+ T cell anti-tumor function, highlighting a therapeutic avenue in improving T cell-based immunotherapy (153). Atorvastatin, one of the classic cholesterol-lowering drugs, down-regulates co-inhibitory receptors expression and increases IL-2 secretion by inhibiting the ras-activated MAPK and PI3K-Akt pathways and subsequent mTOR signaling to ameliorate activated T-cell function (154). In contrast, inhibiting cholesterol esterification with the increase in plasma membrane cholesterol level is a proven potentiated effector function and enhances proliferation of CD8+ TILs, but not CD4+ TILs, a result of the enhanced T-cell receptor clustering as well as the more efficient formation of the immunological synapse (155). Yang W et al. demonstrated that a combined therapy of avasimibe, the inhibitor of Acyl-coenzyme A:cholesterol acyltransferase1 (ACAT1) which is a crucial cholesterol esterification enzyme (156), with an anti-PD-1 antibody offers better efficacy than monotherapies in controlling tumor progression of melanoma-bearing mice (155). The combination therapy of targeting lipid metabolism with ICIs is under further study.

Moreover, more attention has been focused on sphingolipid metabolism in tumor biological behavior, immune escape and anticancer therapy. Sphingolipids, including the two central bioactive lipids, ceramide and sphingosine-1-phosphate (S1P), have opposing roles in sustaining cancer cell survival (157). Neutral sphingomyelinase 2 (nSMase2) with *SMPD3* encoding catalyzes the breakdown of sphingolipid to produce the anti-tumor ceramide linked to apoptosis, growth arrest and chemotherapeutic response (158), accumulates TH1 polarization and CD8+ TILs with increased IFN-γ and chemokine C-X-C motif ligand 9 (CXCL9), and even increases anti-PD-1 efficacy in murine models of melanoma and breast cancer (159). Nonetheless, nSMase2 is generally downregulated in many cancers (158). Sphingosine kinase (SK) catalyzes the phosphorylation of sphingosine to S1P. SK behaves as an immune escape lipid kinase with increased expression of immunosuppressive factors in many cancers. Increased expression of SK1 in tumor cells is significantly associated with shorter survival in metastatic melanoma patients treated with anti-PD-1, and targeting SK1 enhances the responses to ICIs in murine models of melanoma, breast and colon cancer with limited Tregs infiltration (160). These preclinical studies provide a solid foundation for further clinical trials to overcome ICI resistance by targeting sphingolipid metabolism.

## Nucleotide Metabolism Reprogramming in Cancer Cells With Immune Cells and Their Effects on the Expression of Immune Checkpoints

The process of nucleotide synthesis includes *de novo* synthesis and salvage pathway. *De novo* synthesis, as the main synthesis pathway, refers to the process of forming ribonucleotides through a series of enzymatic reactions with phosphoribose, amino acids, one carbon unit and CO2. Then, the relevant deoxyribonucleotides diphosphate (dNDP), as the precursors of deoxyribonucleotide triphosphate (dNTP) participating in DNA synthesis, are produced by ribonucleotides catalyzed by ribonucleotide reductase (RNR). Acetylation and deacetylation of the ribonucleotide reductase small subunit M2 (RRM2) act as a crucial switch that impacts dNTP synthesis and DNA replication fork progression (161). Compared to normal cells, cancer cells have a greater demand for dNTP pool to satisfy the proliferative and invasive activities (162). And the increased nucleotide *de novo* synthesis drives the metastasis and stemness of cancer cells with more cyclic guanosine monophosphate (cGMP) (163). Required nutrient deficiency competing with cancer cells, such as one-carbon unit and aspartate, was also reported to impair the immunity of the TME, such as activation of naive T cells and the expansion function of the effector T cell (128, 164). Remarkably, enriched nucleosides or enhanced RRM2 expression promote tumorigenesis by suppressing oncogene-induced stable associated cell growth arrest, and dNTP repression with RRM2 knockdown could inhibit the growth of cancer cells (165). Additionally, statistical analysis demonstrated that high expression of RRM2 was relevant to immunosuppressive TME (166). Although several generations of RNR inhibitors have been developed in anti-tumor therapy (167), there has been a lack of exciting performance, especially in combination with ICIs. The dihydroorotate dehydrogenase (DHODH) is the enzyme responsible for the fourth step of *de novo* pyrimidine biosynthesis and coupled to the mitochondrial electron transport chain. Although DHODH is not known to be mutated or over-expressed in patients with cancer, DHODH inhibitors are able to shrink tumor burden and improve survival by inducting leukemic cells differentiation in preclinical AML models (168). Unexpectedly, a phase I/II clinical trial on the combination of leflunomide, a human DHODH inhibitor, and the BRAF inhibitor vemurafenib in metastatic melanoma patients with BRAFV600 mutations was terminated due to serious AEs (NCT01611675) (168).

Under endogenous replication stress, DNA breakage and DNA damage response and repair (DDR) play essential roles in tumorigenesis, tumor progression and immune regulation (169). DDR gene alternations are associated with increased TILs, higher genomic instability, increased tumor mutation load (TMB) and improved clinical outcomes in cancer (170). It was reported that DDR alterations are associated with higher ORR, longer PFS and OS in urothelial carcinoma and NSCLC patients treated with PD-1/PD-L1 blockade (170, 171). Besides, Tu X et al. demonstrated that intracellular PD-L1 regulates the DDR by binding and stabilizing the mRNAs of DNA damage genes, and a novel PD-L1 antibody, H1A, can sensitize cancer to DNA-damaging therapy, radiation or chemotherapy, by promoting PD-L1 degradation (172). Moreover, Sen T et al. demonstrated that targeting DDR proteins with poly ADP-ribose polymerase (PARP) or checkpoint kinase 1 significantly increases the expression of PD-L1, remarkably potentiated the anti-tumor effect of PD-L1 blockade and augmented cytotoxic T-cell infiltration in SCLC mouse models with the activation of STING/TBK1/IRF3 innate immune pathway (173). Although the ORR of ICIs in ovarian cancer is only 8-9%, preclinical studies have shown that combining ICIs augment the anti-tumor effects of DDR inhibitor olaparib (174). A phase 1a/b clinical trial showed that pamiparib, an oral PARP 1/2 inhibitor, with tislelizumab, a humanised anti-PD-1 monoclonal antibody, was generally well tolerated and had an ORR of 20% in patients with a variety of advanced solid tumors, including ovarian cancer (175). Furthermore, TOPACIO trial, a phase I/II clinical trial of the PARP inhibitor niraparib in combination with the anti-PD-1 antibody pembrolizumab in recurrent ovarian cancer frequently develop resistance to platinum-based chemotherapy, demonstrated an 18% ORR and a clinical benefit rate of 65%, clearly exceeding the expected activity of niraparib or pembrolizumab as monotherapies in recurrent platinum-resistant ovarian cancer (176).

Other than mutations of oncogenes and tumor suppressors, epigenetic reprogramming, such as histone modifications, DNA methylation, and noncoding RNAs, also drive the phenotypic changes of tumor cells to escape from immune surveillance and construct an immunosuppressive TME (177). In this way, a rational combination of PD-L1/PD-1 blockade and epigenetic agents may offer great potential in retraining the immune system and improving clinical outcomes of anti-tumor treatment. Some phase I/II clinical trials on the combination of ICIs and epigenetic agents among different cancer types are designed and ongoing (177).

## Aberrant Mitochondrial Biogenesis in Cancer Cells With Immune Cells and Their Effects on the Expressions of Immune Checkpoints

ATP, as the dominant energy provider which plays a central role in energy generation, transformation, storage, utilization and other biological activities, is mainly produced by the catabolic metabolism of glucose, lipids and amino acids with TCA and OXPHOS. The protons and electrons removed from the metabolites are present as reducing equivalents (NADH+H^+^, FADH2) in mitochondria, which are pumped into the cytosol by an electron transport chain consisting of four complexes, forming an electrochemical gradient of H^+^ across the mitochondrial membrane, while the ATP synthase utilizes the potential energy released during its reflux down the gradient to drive ATP release. Although glycolysis predominates in the metabolic reprogramming of cancer cells, OXPHOS is also enhanced in tumor cells, and some compounds targeting OXPHOS to suppress tumors have been studied (178, 179). An example is metformin, which could decrease cancer cell proliferation by decreasing the NAD+/NADH ratio and inhibiting aspartate production (180), and stimulates the proliferation of CD8+ TILs with the production of mitochondrial ROS in an NF-E2-related factor 2 (Nrf2)/mTORC1/p62 pathway (181). It was also reported that metformin could further improve intratumoral T-cell function and tumor clearance when combined with ICIs (62, 181, 182). Phase II clinical trial showed metformin therapy was associated with better-than-expected OS on non-diabetic patients with advanced-stage ovarian cancer (183). The median PFS of 18.0 months and OS of 57.9 months compare favorably to other clinical trials with similar patient populations and historical expectations (183). The clinical trial focus on the combination therapy of Nivolumab with metformin for refractory or recurrent solid tumors is ongoing (184). Another example is IACS-010759, which has been reported to emphatically inhibit the proliferation of hypoxic tumor cells by interfering with the functions of mitochondrial NADH-ubiquinone oxidoreductase (complex I) without exhibiting cytotoxicity at tolerated doses in normal cells (185). And preclinical trial demonstrated that combined IACS-010759 and radiotherapy promoted anti-tumor effects in the PD-1-resistant model, though not in the sensitive model (186). Unfortunately, there has been no relevant clinical trial.

Different immunocyte populations have different metabolic educations in the metabolically reprogrammed TME. Quiescent T cells and Tregs depend on OXPHOS for energy production, while effector T cells mainly rely on glycolysis for proliferation (187). Besides, effector memory and central memory CD4+ T cells have elevated glycolysis and OXPHOS compared to naive T cells. When naive T cells respond to TCR stimulation, robust and rapid glycolysis and OXPHOS occur, accompanied by Akt or STAT5 signaling activation (188). Elevated OXPHOS also participates in immunoregulation with increased MHC presentation (189). Compared to pro-inflammatory M1 macrophages, immunosuppressive M2 macrophages are more dependent on OXPHOS (72). The immunosuppressive TME represses mitochondrial biogenesis (190), whereas 4-1BB costimulation increases mitochondria numbers in CD8+ T cells (191). Also, PD-1 engagement in CD8+ T cells would trigger a suppressed OXPHOS process with decreased mitochondrial cristae and unexpectedly greater assembly of respiratory supercomplexes different from resting cells and activated T cells (31). These results suggest that mitochondrion is the primary target of immune checkpoints engaged with the activated or inhibitory activity. Nevertheless, the role of OXPHOS in ICIs resistance is controversial. High-resolution mass spectrometry revealed higher OXPHOS in responders with TIL-based or anti-PD-1 immunotherapy than non-responders in melanoma patients (149). Alternatively, Chen D et al. considered the PD-1-resistant model, which seemed to utilize OXPHOS to a significantly greater extent than the PD-1-sensitive NSCLC murine model (186). They further demonstrated that the combination of OXPHOS inhibitor, IACS-010759 and radiotherapy promoted anti-tumor effects in the PD-1-resistant model, but not in the sensitive tumor-burdened animal model (186). Moreover, Jia L et al. tried to enhance the immunity of TILs by balancing the glycolysis and OXPHOS of tumor cells, and this balancing strategy provides a more reliable immune-boosting strategy to PD-L1 silencing than complete glycolysis inhibition (179).

Under the stress of hypoxia, inflammation and deficient nutrient, ATP is transferred to the extracellular space (192). Accumulated ATP in the TME is metabolized into immunosuppressive adenosine by upregulated ectonucleotidases in cancer. Increased adenosine binding to its receptors block proliferation of T cells, promote immunosuppressive Tregs, prevent the cytotoxic activity of NK cells and M1 macrophages, and induce the upregulation of co-inhibitory molecules (PD-1 and CTLA-4) and tolerogenic cytokines (193–195). It has been reported that the increased ectonucleotidase and adenosine are related to hypoxia exposure in many cancer cells (196, 197). Remarkably, adenosine drives the overexpression of LDH5, a crucial mediator promoting anaerobic glycolysis, creating a vicious cycle because the large amount of ATP produced by glycolysis feeds the production of adenosine in an ectonucleotidase-rich TME (198). Serra S et al. have shown that the A2A adenosine receptor blockade counteracts these effects, making leukemic cells more susceptible to pharmacological agents while restoring immune competence and T-cell proliferation *in vitro* studies (197). Moreover, dual blockade of PD-1 and A2A significantly enhances the cytotoxicity of CD8+ TILs, in turn inhibiting tumor progression and prolonging tumor-burden mice’s survival (199).

## Biomarkers in ICIs Therapy From Reprogramming Metabolism

Although there have been exciting reports about the roles of ICIs in anti-tumor therapy, the response rate is not satisfactory. Low ORR, short survival and high recurrence have been reported in many cancer patients with ICIs treatment. Finding the appropriately predictive and prognostic biomarker is important in anti-tumor immunotherapy. Currently, the intensity and density of PD-1/PD-L1 expression in tumor tissue is commonly considered to be the standard biomarker for predicting the efficacy of immunotherapy. However, some cancer patients with high PD-(L)1 expression have been reported to have an inadequate response or even resistance to anti-PD-(L)1, whereas some patients with low PD-(L)1 expression have a strong response (200). It is necessary to search for more precise prognostic markers in the anti-tumor immunotherapy.

Based on the crucial role of the reprogramming metabolism in tumor progression and immunosuppression, many metabolism-related molecules have been investigated for their predictive and prognostic values in immunotherapy. TMB is reportedly an effective biomarker in many tumors with ICIs and have been applied in clinical studies (201, 202). Compared to TMB and aneuploidy, glycolytic activity is a stronger and more consistent predictor of immune signatures in a diverse range of cancers (203). Notably, Jiang Z et al. showed that highly glycolytic tumors exhibited a better immunotherapy response and favorable survival in the immunotherapy setting (203). LDH and tumor acidity are also reported as predictive and prognostic markers for immunotherapy (204). Besides, Xue G et al. even showed a better predictive and prognostic value of the CpG-based model than TMB in cancer patients (205). In addition, circulating tumor DNA seem to have predictive and prognostic values in anti-tumor immunotherapy (206).

## Discussion

Immunometabolism has attracted increasing research interest on anti-tumor therapeutic applications recently (207). Metabolic reprogramming, hypoxia, genetic mutation and immune regulation control each other interdependently and collectively, contributing to tumor progression and immune compromise (208). Under the education of oncogenes and tumor suppressors, tumor cells and immune cells reprogram the metabolism with key molecular alterations to accommodate tumor progression and immune suppression (**Table 2**). Targeting altered metabolism within tumor cells provides the possibility of improving the efficacy of immunotherapy. In this article, we have demonstrated the reprogramming metabolism in cancer cells and immune cells, and described the regulatory mechanisms of immune checkpoints involved. More importantly, we tried to cite preclinical and clinical trial evidence to comprehensively demonstrate that combined targeting metabolic therapy with ICIs may enhance anti-tumor abilities of immune cells, overcome drug resistance, and even prolong the PFS/OS of cancer patients, which provide a new perspective in anti-tumor immunotherapy. In addition to this, we sought to find biomarkers with greater prognostic value for ICIs treatment from a metabolic perspective.

**Table 2:** The effect of key metabolic targets on cancer cells and immune cells in the TME.

Metabolic pathways	Effect molecule	Effect cell types	Up or down regulated	Pro or anti-tumor
Hypoxia	HIFs	Cancer cells	Up-regulated	Pro-tumor/Immunosuppression
Glycolysis	PKM2	Cancer cells, Macrophages, DCs, T cells	Up-regulated	Pro-tumor/Immunosuppression
	PEP	T cells	Down-regulated	Immune activation/Anti-tumor
	GSK3	Cancer cells	Up-regulated	Immunosuppression/Pro-tumor
	FBP1	Cancer cells	Down-regulated	Anti-tumor
Amino acid metabolism	Glutamine	Cancer cells	Up-regulated	Pro-tumor
	Glutamine	Immune cells	Down-regulated	Immune activation
	Arginine	Cancer cells	Up-regulated	Pro-tumor/Immunosuppression
	Arginine	Immune cells	Down-regulated	Immune activation
	IDO	Cancer cells, Immune cells, Immune cells	Up-regulated	Immunosuppression/Pro-tumor
	SAM	T cells	Down-regulated	Immune activation/Anti-tumor
	SLC43A2	Cancer cells	Up-regulated	Immunosuppression/Pro-tumor
Lipid metabolism	SREBPs	Cancer cells	Up-regulated	Pro-tumor
	SREBPs	Tregs	Up-regulated	Immunosuppression/Pro-tumor
	FATP2	MDSCs	Up-regulated	Immunosuppression/Pro-tumor
	Lipid	DCs	Up-regulated	Immunosuppression/Pro-tumor
	CD36	CD8+ TILs	Up-regulated	Immunosuppression/Pro-tumor
	Cholesterol	CD8+ TILs	Up-regulated	Immunosuppression/Pro-tumor
	nSMase2	Cancer cells	Down-regulated	Anti-tumor/Immune activation
	SK	Cancer cells	Up-regulated	Pro-tumor/Immunosuppression
Nucleotide metabolism	cGMP	Cancer cells	Up-regulated	Pro-tumor
	dNTP	Cancer cells	Up-regulated	Pro-tumor
	RRM2	Cancer cells	Up-regulated	Pro-tumor/Immunosuppression
Mitochondrial biogenesis	Ectonucleotidases	Cancer cells, Immune cells	Up-regulated	Immunosuppression/Pro-tumor

HIFs, hypoxia-inducible factors; PKM2, Pyruvate kinase isoform M2; PEP, phosphoenolpyruvate; GSK3, Glycogen synthase kinase 3; FBP1, fructose-1, 6-biphosphatase; αKG, α-ketoglutarate; IDO, Indoleamine 2,3-dioxygenase; SREBPs, sterol regulatory element-binding proteins; FATP2, fatty acid transport protein 2; nSMase2, Neutral sphingomyelinase 2; SK, sphingosine kinase; cGMP, cyclic guanosine monophosphate; dNTP, deoxyribonucleotide triphosphate; RRM2, ribonucleotide reductase small subunit M2; DCs, dendritic cells; Tregs, regulatory T cell; TH1, T helper 1; MDSCs, myeloid-derived suppressor cells; PD-L1, programmed death ligand 1; CD47, cluster of differentiation 47; HLA-G, human leukocyte antigen G; TILs, tumor infiltrating lymphocytes; FAO, fatty acid oxidation; mTORC1, mammalian target of rapamycin complex 1; NO, nitric oxide; TME, tumor microenviroment; GM-CSF, granulocyte macrophage-colony stimulating factor; MHC, major histocompatibility complex; XBP1, X-box binding protein 1; IFN-γ, interferon-γ; ATP, adenosine triphosphate.

Although ICIs have shown an impressive performance in the treatment of solid tumors, their ORR is around 20-40%. It is worth exploring how to improve its efficiency in anti-tumor treatment, overcome drug resistance and prolong the survival of cancer patients. A rising number of efforts have attempted to tip the balance of immune compromise to anti-tumor immunity by ICIs combined with chemotherapy, radiotherapy (209), targeted agents (210), innate immunity modulating drugs (211), even anti-platelet (212) and other therapies. Chemotherapy potentiates the immunogenicity of tumor cells to enhance immune recognition and immune elimination, and immunotherapy increases the sensitivity of tumor cells to chemotherapy (213). As shown in phase 3 clinical trial of locally advanced or metastatic non-squamous NSCLC patients without *EGFR* or *ALK* genomic aberration, NCT03607539, Sintilimab (anti-PD-1) plus pemetrexed and platinum had a longer median PFS (8.9 *versus* 5.0 months) and a better ORR (51.9% *versus* 29.8%) than that in the placebo plus pemetrexed and platinum group (214). In addition to destroying tumor cells to release antigens for enhanced antigen presentation, radiotherapy also enhances anti-tumor immunity by upregulating MHC-I expression and the cGAS-STING pathway (215), increasing TCR density (216) and the abscopal effect (209). Pacific trial, a phase III clinical trial on unresectable NSCLC patients, demonstrated longer PFS (17.2 *vs* 5.6 months), OS (28.3 *vs* 16.2 months) and a higher ORR (30% *vs* 17.8%) in patients treated with anti-PD-L1 Duvalumab within 1-14 days of completing chemoradiotherapy (217). CheckMate 067 further demonstrated a significantly enhanced ORR in melanoma patients treated with Nivolumab plus Ipilimumab (58%) with no apparent loss of quality of life, compared to Ipilimumab (19%), Nivolumab (45%) alone (218). NSCLC patients harboring *EGFR* mutations or *ALK* rearrangements are commonly thought to be associated with a low ORR to PD-1/PD-L1 inhibitors. However, a retrospective analysis revealed that the proportion of tumors with high-expressed PD-L1 was higher among T790M-negative patients than among T790M-positive patients after disease progression during EGFR-TKI treatment (219). This suggests the potential of combining ICIs with EGFR-TKIs to improve efficacy and overcome drug resistance.

Nevertheless, the metabolic reprogramming of tumor cells and immune cells is one of the crucial obstacles to immunotherapy, and targeting reprogrammed metabolites provides a new perspective to improve the efficacy of ICIs in addition to chemotherapy, radiotherapy and targeted therapy. Many preclinical and phase I/II clinical trials have demonstrated promising performance of the combination of targeting metabolism with immunotherapy in overcoming drug resistance and minimizing tumor burden (**Table 3**). In particular, PARP inhibitors, ADI-PEG 20, IDO inhibitor and metformin have shown exciting performance in combination with ICIs in clinical trials. Furthermore, phase III clinical trials with extended samples on ORR, PFS and OS still need to be explored. Additionally, while pursuing an improved benefit rate, the combination therapy should pay attention to prevent the occurrence of serious AEs. The metabolic reprogramming process is integral and cannot be separated from each other completely. Although targeted tumor metabolic therapy provides access to more nutrients and oxygen to confers immune cell function, the metabolic pathways within tumor cells and immune cells are frequently similar, making tumor cells damaged while immune cells are injured by targeting metabolic signals. Such as rapamycin, which interferes with the proliferation, growth and survival of cancer cells as well as having immunosuppressive effects (220).

**Table 3:** Preclinical and clinical trials of drugs inhibiting metabolic targets with ICIs in anti-tumor therapy.

Targeted metabolic pathway	Preclinical/Clinical trial	Drug	Targeting models/patients	Result of the trial	Title
Hypoxia	Preclinical trial	Metformin+ PD-1 blockade	Tumor-bearing murine models (B16 melanoma, MC38 colon adenocarcinoma)	Although metformin monotherapy had little therapeutic benefit in highly aggressive tumors, combination of metformin with PD-1 blockade resulted in improved intratumoral T-cell function and tumor clearance.	Efficacy of PD-1 Blockade Is Potentiated by Metformin-Induced Reduction of Tumor Hypoxia (Scharping NE et al, 2017, Ref 62).
	Phase II Clinical trial (NCT03709147)	platinum + pemetrexed + pembrolizumab +metformin	Patients with advanced LKB1-inactive lung adenocarcinoma	Ongoing.	Exploiting Metformin Plus/Minus Cyclic Fasting Mimicking Diet (FMD) to Improve the Efficacy of First Line Chemo-immunotherapy in Advanced LKB1-inactive Lung Adenocarcinoma.
Glycolysis	Preclinical trial	Bicarbonate+ anti-CTLA-4 or anti-PD1	Tumor-bearing murine models (B16 melanoma)	The combination of bicarbonate therapy neutralizing tumor acidity with anti-CTLA-4 or anti-PD1 improved antitumor responses.	Neutralization of Tumor Acidity Improves Antitumor Responses to Immunotherapy (Philon Thomas S. 2019, Ref 80).
	Preclinical trial	FX-11 (LDH inhibitor) +anti-PD-1	Tumor-bearing murine models (TNBC)	The inhibition of LDH could enhance tumor response to anti-PD-1 immunotherapy in TNBC murine models.	Metabolic-Pathway-Based Subtyping of Triple-Negative Breast Cancer Reveals Potential Therapeutic Targets (Gong Y, et al, 2021, Ref 83).
Arginine	Preclinical trial	ADI-PEG 20 (PEGylated arginine deiminase)+anti-PD-1+anti-PDL1	Tumor-burdened mice	The combination of ADI-PEG 20, anti-PD-1 and anti-PD-L1 results in a more powerful anti-tumor therapy when compared to monotherapy.	PEGylated arginine deiminase can modulate tumor immune microenvironment by affecting immune checkpoint expression, decreasing regulatory T cell accumulation and inducing tumor T cell infiltration (Brin E et al, 2017, Ref 110).
	Phase 1b Clinical trial	ADI-PEG 20 + Pembrolizumab (anti-PD-1)	Patients with advanced solid cancers	ADI-PEG 20 combined with pembrolizumab demonstrated well-tolerated drug AEs and a promising disease control rate in advanced solid cancers.	Phase 1b study of pegylated arginine deiminase (ADI-PEG 20) plus Pembrolizumab in advanced solid cancers. (Chang KY, et al, 2021, Ref 111)
	Preclinical trial	PT1001B (arginine methyltransferase inhibitor)+ICIs	Mouse model (pancreatic cancer)	The combination of protein arginine methyltransferase inhibitor with ICIs enhanced the anti-tumor immunity with increased CD8+ TILs.	Combining protein arginine methyltransferase inhibitor and anti-programmed death-ligand-1 inhibits pancreatic cancer progression. (Zheng NN et al, 2020, Ref 112)
Tryptophan	Preclinical trial	IDO inhibitor +ICIs	IDO knockout/wild type mice burdened with B16 melanoma.	The antitumor effect of anti-CTLA-4 or anti-PD-L1/anti-PD-1 was significantly increased in IDO-deficient melanoma bearing mice.	Indoleamine 2,3-dioxygenase is a critical resistance mechanism in antitumor T cell immunotherapy targeting CTLA-4 (Holmgaard RB et al, 2013, Ref 113).
	Phase I/II Clinical trial (NCT02073123)	Indoximod (IDO inhibitor) + Ipilimumab (anti-CTLA-4)	Advanced (III/IV stage) or metastatic melanoma patients	Completed. (No Study Results Posted)	A Phase 1/2 Study of the Concomitant Administration of Indoximod Plus Immune Checkpoint Inhibitors for Adult Patients With Advanced or Metastatic Melanoma.
	Phase I/II Clinical trial (NCT03277352)	INCAGN01876 (IDO inhibitor) +Pembrolizumab (anti-PD-1)	Patients with advanced or metastatic malignancies	Terminated.	A Phase 1/2 Safety and Efficacy Study of INCAGN01876 in Combination With Immune Therapies in Subjects With Advanced or Metastatic Malignancies.
	Phase I Clinical trial (NCT04047706)	IDO1 Inhibitor BMS-986205 +Nivolumab (anti-PD1)	Glioblastoma patients	Ongoing.	Combination of Checkpoint Inhibition and IDO1 Inhibition Together With Standard Radiotherapy or Chemoradiotherapy in Newly Diagnosed Glioblastoma.
Methionine	Preclinical trial	SLC43A2 inhibitor+anti-PD-1	Tumor-bearing mice	The combination treatment of SLC43A2 inhibitor and anti-PD-1 inhibited tumor growth and enhanced cytokine production by CD8+TILs in tumor-bearing mice.	Cancer SLC43A2 alters T cell methionine metabolism and histone methylation (Bian Y et al, 2020, Ref 125).
	Preclinical trial	SAM +ICI	Melanoma mouse model	The combination of SAM and ICI effectively block melanoma by alteration of key genes and pathways implicated in cancer and immune responses compared to monotherapy, providing the rationale for initiating clinical trials with SAM and ICI.	Enhanced Anticancer Effect of a Combination of S-adenosylmethionine (SAM) and Immune Checkpoint Inhibitor (ICPi) in a Syngeneic Mouse Model of Advanced Melanoma. (Mehdi A, et al., 2020, Ref 124).
Cystine-glutamate exchange (xCT)	Preclinical trial	Sulfasalazine (xCT inhibitor)+ PD-1/PD-L1 blockade	Tumor-bearing mice with B16 melanoma.	The inhibition of xCT blunted the efficacy of PD-1/PD-L1 blockade through upregulating PD-L1 expression, and thus exosomes carrying large amounts of PD-L1 secreted from melanoma cells induced macrophage M2 polarization and eventually induced anti-PD-1/PD-L1 therapy resistance.	Inhibition of xCT suppresses the efficacy of anti-PD-1/L1 melanoma treatment through exosomal PD-L1-induced macrophage M2 polarization (Liu N et al, 2021, Ref 127).
Lipid	Preclinical trial	Lipofermata +anti-PD-L1	Tumor-bearing mice	The blockage of FATP2 expression in MDSCs by lipofermata decreased lipid accumulation, decreased ROS, blocked immunosuppressive activity, lower PD-L1 expression on CD8+ TILs, and even enhanced anti-PD-L1 tumor immunotherapy.	Regulation of ROS in myeloid-derived suppressor cells through targeting fatty acid transport protein 2 enhanced anti-PD-L1 tumor immunotherapy (Adeshakin AO et al, 2021, Ref 136).
	Preclinical trial	CD36 inhibitor+ anti-PD-1	Tumor-bearing mice with B16 melanoma	Blocking CD36 or inhibiting ferroptosis in CD8+ T cells effectively restored their antitumor activity and possessed greater antitumor efficacy in combination with anti-PD-1 antibodies.	CD36-mediated ferroptosis dampens intratumoral CD8+ T cell effector function and impairs their antitumor ability (Ma X et al, 2021, Ref 141)
	Preclinical trial	Avasimibe (inhibitor of ACAT1)+ anti-PD-1	Melanoma-bearing mice	A combined therapy of avasimibe with an anti-PD-1 antibody showed better efficacy than monotherapies in controlling tumor progression.	Potentiating the antitumor response of CD8(+) T cells by modulating cholesterol metabolism (Yang W et al, 2016, Ref 155).
	Preclinical trial	SK inhibitor +ICIs	Murine models of melanoma, breast and colon cancer.	Targeting SK1 marked enhances the responses to ICIs in murine models of melanoma, breast and colon cancer.	Resistance of melanoma to immune checkpoint inhibitors is overcome by targeting the sphingosine kinase-1 (Imbert C et al, 2020, Ref 160).
Nucleotide	Preclinical trial	PARP or checkpoint kinase 1+PD-L1 blockade	SCLC mouse models	Targeting DDR proteins significantly increase the expression of PD-L1 and remarkably potentiated the anti-tumor effect of PD-L1 blockade and augmented cytotoxic T-cell infiltration.	Targeting DNA Damage Response Promotes Antitumor Immunity through STING-Mediated T-cell Activation in Small Cell Lung Cancer (Sen T et al, 2019, Ref 173).
	Phase 1a/b clinical trial	Pamiparib (an oral PARP 1/2 inhibitor)+ Tislelizumab (anti-PD-1)	Patients with a variety of advanced solid tumors, including ovarian cancer	Pamiparib, an oral PARP 1/2 inhibitor, with tislelizumab, a humanised anti-PD-1 monoclonal antibody, was generally well tolerated and had an ORR of 20% in patients with a variety of advanced solid tumors, including ovarian cancer.	Pamiparib in combination with tislelizumab in patients with advanced solid tumors: results from the dose-escalation stage of a multicentre, open-label, phase 1a/b trial. (Friedlander M, et al, 2019, Ref 175)
	Phase I/II Clinical trial TOPACIO trial	Niraparib (PARP inhibitor)+ Pembrolizumab (anti-PD-1)	Recurrent platinum-resistant ovarian cancer patients	Niraparib in combination with pembrolizumab demonstrated an 18% ORR and a clinical benefit rate of 65%, clearly exceeding the expected activity of niraparib or pembrolizumab as monotherapies in recurrent platinum-resistant ovarian cancer.	Single-Arm Phases 1 and 2 Trial of Niraparib in Combination With Pembrolizumab in Patients With Recurrent Platinum-Resistant Ovarian Carcinoma. (Konstantinopoulos PA, et al, 2019, Ref 176)
	Phase II Clinical trial (NCT03786796)	Olaparib	Patients with metastatic renal cell carcinoma who have had prior treatment with at least one immune checkpoint inhibitor or anti-VEGF therapy.	Ongoing.	Phase II Study of Olaparib in Metastatic Renal Cell Carcinoma Patients Harboring a BAP-1 or Other DNA Repair Gene Mutations (ORCHID).
Mitochondrial biogenesis	Preclinical trial	IACS-010759 (OXPHOS inhibitor)+ radiotherapy	Tumor-burdened mice	The combination of OXPHOS inhibitor and radiotherapy promoted antitumor effects in the PD-1-resistant model, but not in the sensitive tumor-burdened animal model.	Combination treatment with radiotherapy and a novel oxidative phosphorylation inhibitor overcomes PD-1 resistance and enhances antitumor immunity (Chen D et al, 2020, Ref 186).
	Preclinical trial	A2A adenosine receptor blockade+PD-1 blockade	Tumor-burdened mice	The dual blockade of PD-1 and A2A significantly enhanced the cytotoxicity of CD8+ TILs to inhibit tumor progression and prolong the tumor-burden mice’s survival.	Adenosine Receptor 2A Blockade Increases the Efficacy of Anti-PD-1 through Enhanced Antitumor T-cell Responses (Beavis PA et al, 2015, Ref 200).

Details on clinical trial design can be found on https://clinicaltrials.gov/.

IDO, Indoleamine 2,3-dioxygenase; LDH, lactate dehydrogenase; FATP2, fatty acid transport protein 2; ACAT1, Acyl-coenzyme A:cholesterol acyltransferase 1; SK, sphingosine kinase; DDR, DNA damage response and repair; OXPHOS, oxidative phosphorylation; ICIs, Immune checkpoint inhibitors; PD-1, programmed cell death protein 1;SCLC, small cell lung cancer; ROS, reactive oxygen species; CTLA-4, cytotoxic T-lymphocyte antigen-4; TNBC, triple negative breast cancer; MDSCs, myeloid-derived suppressor cells; IL-2, interleukin-2; PARP, poly ADP-ribose polymerase.

Among the reprogramming metabolic processes of cancer cells and immune cells, mTOR signaling seems to play a vital role in bridging metabolism and immunity. mTOR signaling controls cellular metabolism, immune cell differentiation, and effector function (131). The PI3K-mTOR-AKT signaling pathway not only promotes tumor cell growth and proliferation in the majority of cancers, but also controls the activation and polarization of macrophages, and decreases T-cell infiltration (131). Meanwhile, the mTOR signaling is also involved in immune checkpoint expression and downstream signaling in tumor cells and immune cells. It mediated the co-inhibitory checkpoints expression under the cytokines stimulation and participates in the PD-1/PD-L1 downstream signaling to reprogram cellular metabolism. mTOR signaling is also involved in the reprogramming lipid, amino acids and nucleotide metabolism in cancer cells and immune cells to promote tumor cell progression and escape immunological surveillance. Further studies on the role of the mTOR signaling in controlling the immunometabolism in the TME will better facilitate the efficacy and minimize the side effects of targeting metabolism in combination with ICIs.

In addition to the metabolic reprogramming of tumor cells and immune cells to manipulate the tumor-immune set point in TME, the metabolism of the gut microbiome has received increasing attention on the anti-tumor immune regulation. Microbiota-derived short-chain fatty acids, such as butyrate, promote the formation of long-term CD8+ memory T cells and modulate Tregs function (207, 220). Furthermore, in melanoma patients, a favorable gut microbiome combined with anti-PD-1 resulted in enhanced anti-tumor responses associated with improved Teff cell function (221). In this regard, supporting gut microbiota-related metabolism may be an effective adjuvant to anti-tumor immunotherapy.

Not all metabolic changes equally contribute to promoting the malignant transformation and progression of tumor cells. These metabolic changes can be divided into three categories: transforming activities, enabling activities and neutral activities (208). Only a tiny portion of metabolic activities are transforming activities that directly contribute to tumorigenesis and antagonize anti-tumor activities. Most metabolic reprogramming are enabling activities that change within tumor cells but are not involved in cell transformation, while neutral activities are dispensable to tumor cell progression. Nonetheless, neutral activities reportedly have predictive and prognostic values. Intriguingly, even the same kind of tumor cells may have different metabolic remodeling, thus even the same targeting metabolic therapy may have different effects. Gong Y et al. demonstrated that there are three heterogeneous metabolic-pathway-based subtypes (MPSs) with distinct metabolic features: MPS1, the lipogenic subtype with upregulated lipid metabolism; MPS2, the glycolytic subtype with upregulated carbohydrate and nucleotide metabolism; and MPS3, the mixed subtype with partial pathway dysregulation, which exists in TNBC patients with distinct prognoses, molecular subtype distributions and genomic alterations (83). Furthermore, even the same kind of metabolic reprogramming in the TME has different immune effects on different immune cells, and these differences manifest the charms of tumor heterogeneity. In an era of individualized precision treatment, providing the right personalized treatment to the right patients at the right time for optimizing benefits for each person requires close cooperation between various fields.

## Author Contributions

YX and LH conceived this review. This review were performed by YX, QF, LH, and JH collectively. YX wrote the manuscript. LH polished this review. All authors contributed to the article and approved the submitted version.

## Funding

This work was financially supported by the PhD Research Startup Foundation of Liaoning Province, China (No. 2020-BS-039).

## Conflict of Interest

The authors declare that the research was conducted in the absence of any commercial or financial relationships that could be construed as a potential conflict of interest.

## Publisher’s Note

All claims expressed in this article are solely those of the authors and do not necessarily represent those of their affiliated organizations, or those of the publisher, the editors and the reviewers. Any product that may be evaluated in this article, or claim that may be made by its manufacturer, is not guaranteed or endorsed by the publisher.

## Glossary

**Table:**

ACAT	Acyl-coenzyme A:cholesterol acyltransferase
acetyl-CoA	acetyl coenzyme A
ADI-PEG 20	PEGylated arginine deiminase
AEs	adverse events
ALK	Anaplastic Lymphoma Kinase
APCs	Antigen-presenting cells
ARGs	arginases
ASCT2	alanine
serine	cysteine system amino acids transporter 2
ASS1	argininosuccinate synthetase 1
ATP	adenosine triphosphate
αKG	α-ketoglutarate
CAIX	carbonic anhydrase IX
CAT	cationic amino acid transporters
CD47	cluster of differentiation 47
cGMP	cyclic guanosine monophosphate
CTLA-4	cytotoxic T-lymphocyte antigen-4
CXCL9	Chemokine (C-X-C motif) ligand 9
DDR	DNA damage response and repair
DCs	dendritic cells
DHODH	dihydroorotate dehydrogenase
dNDP	deoxyribonucleotides diphosphate
dNTP	deoxyribonucleotide triphosphate
EAA	essential amino acid
EGFR	Epidermal Growth Factor Receptor
ER	endoplasmic reticulum
Fabp	fatty acid-binding protein
FAO	fatty acid oxidation
FASN	fatty acid synthetase
FATP2	fatty acid transport protein 2
FBP1	fructose-1, 6-biphosphatase
GLUT	glucose transporters
GM-CSF	granulocyte macrophage-colony stimulating factor
GSK3	Glycogen synthase kinase 3
HCC	hepatocellular carcinoma
HIFs	hypoxia-inducible factors
HK	hexokinase
HLA-G	human leukocyte antigen G
ICIs	Immune checkpoint inhibitors
IDO	Indoleamine 2,3-dioxygenase
IFN-γ	interferon-γ
IL-1β	interleukin-1β
ITIM	immune receptor tyrosine-based inhibitory motif
ITSM	immune receptor tyrosine-based switch motif
JAK	janus kinase
LAG-3	lymphocyteactivation gene 3
LDH	lactate dehydrogenase
MAPK	mitogen-activated protein kinase
MDSCs	myeloid-derived suppressor cells
MHC	major histocompatibility complex
MPSs	metabolic-pathway-based subtypes
mTOR	mammalian target of rapamycin
mTORC1	mammalian target of rapamycin complex 1
NADPH	nicotinamide adenine dinucleotide phosphate
NEAA	nonessential amino acid
NF-κB	nuclear factor κ-B
NO	nitric oxide
NOS	nitric oxide synthase
Nrf2	NF-E2-related factor 2
NSCLC	non-small cell lung cancer
nSMase2	Neutral sphingomyelinase 2
OCT	organic cation transporter
ORR	objective response rate
OS	overall survival
OXPHOS	oxidative phosphorylation
PARP	poly ADP-ribose polymerase
PD-1	programmed cell death protein 1
PD-L1	programmed death ligand 1
PET	[18F]FDG-positron emission tomography
PEP	phosphoenolpyruvate
PFK-1	6-phosphofructokinase-1
PFS	progression-free survival time
PI3K	phosphatidylinositol 3 kinase
PKM2	Pyruvate kinase isoform M2
PP2A	protein phosphatase-2A
PPP	pentose phosphate pathway
RNR	ribonucleotide reductase
ROS	reactive oxygen species
RRM2	ribonucleotide reductase small subunit M2
SAM	S-adenosyl methionine
SCLC	small cell lung cancer
SH2	Src homology 2
SHP2	SH2-containing phosphatase 2
SK	sphingosine kinase
Slc7a5-Slc3a2	solute carrier family
S1P	sphingosine-1-phosphate
SREBP	sterol-regulatory-element-binding proteins
STAT3	signal transducer and activator of transcription 3
TAMs	tumor-associated macrophages
TCA cycle	tricarboxylic acid cycle
TCR	T cell receptor
TH1	T helper 1
TIGIT	T cell immunoreceptor with immunoglobulin and ITIM domains
TILs	tumor infiltrating lymphocytes
TIM-3	T cell immunoglobulin mucin-3
TLR	toll like receptor
TMB	tumor mutation burden
TME	tumor microenvironment
TNBC	triple negative breast cancer
TNF-α	tumor necrosis factor-αTregs, regulatory T cell
Trms	Tissue-resident memory T cells
XBP1	X-box binding protein 1
x-transporter	cystine-glutamate exchange
